# Total calcaneal allograft reconstruction of an Ewing's sarcoma in a child: Outcome and review of the literature

**DOI:** 10.1002/cnr2.1626

**Published:** 2022-05-18

**Authors:** Ferran Torner, Jorge H. Nuñez, Emilio José Inarejos Clemente, Moira Garraus, Mariona Suñol, Aníbal D. Martínez, David Moreno

**Affiliations:** ^1^ Tumor Unit, Department of Traumatology and Orthopedic Surgery Hospital Sant Joan de Deu, Universitat De Barcelona Barcelona Spain; ^2^ Pediatric Department of Traumatology and Orthopedic Surgery Hospital Sant Joan de Deu, Universitat De Barcelona Barcelona Spain; ^3^ Department of Traumatology and Orthopedic Surgery University Hospital of Mutua Terrassa Barcelona Spain; ^4^ Department of Diagnostic Imaging Hospital Sant Joan de Deu, Universitat De Barcelona Barcelona Spain; ^5^ Department of Oncology and Hematology Hospital Sant Joan de Deu, Universitat De Barcelona Barcelona Spain; ^6^ Department of Pathology Hospital Sant Joan de Déu, University of Barcelona Barcelona Spain

**Keywords:** allograft, calcaneus, Ewing's sarcoma; limb salvage surgery, tumor

## Abstract

**Background:**

Ewing's sarcoma rarely presents in bones of the feet. Surgical management usually includes amputation. Limb sparing surgery is anecdotal.

**Case:**

We report the case of a 13‐year‐old boy with an Ewing sarcoma in his calcaneus who had a calcaneal reconstruction with total calcaneus allograft after induction chemotherapy.

**Conclusions:**

At 42 months of follow‐up our patient remains disease free and functionally intact. A review of the exceptional limb salvage procedure options for malignant calcaneus tumor was performed.

## 1 INTRODUCTION

Ewing's sarcoma (ES) is a primary, aggressive malignant tumor.^1^ ES is the second most common malignant bone tumor occurring in children, between 5 and 20 years of age.^2^ Tumors arising in the bones of the foot and ankle are exceedingly rare, accounting for approximately 1% of the primary tumors of the skeleton. According to the literature, primary bone tumors of the calcaneus are less frequent.^3^ Due to this extremely low incidence, current studies are limited, mostly consisting of individual case reports. In sarcomas of the foot, including the calcaneus, below‐knee amputation has been the standard choice of local control due to its poor compartmentalization.^4^, ^5^ Because the standard treatment is amputation, only a few reports that include the surgical limb salvage options have been published.^5^, ^6^

With the development of effective chemotherapy and advanced imaging techniques, limb salvage surgery could be considered the treatment of choice for primary calcaneus malignancy.^5^, ^6^ Different types of calcaneal reconstruction following tumor resection have been reported in few case reports. These techniques included bone allografts, vascularized or pedicle bone autografts, composite allografts with vascularized fibula, and custom‐made calcaneus prosthesis. Each of these surgical methods has their advantages and disadvantages.^7^, ^8^, ^9^, ^10^, ^11^, ^12^, ^13^, ^14^, ^15^, ^16^, ^17^, ^18^ To our knowledge, we present the first case of limb salvage and calcaneus reconstruction after total calcanectomy in a child with Ewing's sarcoma, and long follow‐up. The aim of this report was to analyze the clinical and functional outcomes of this technique in a child. Furthermore, a review of the literature of the exceptional limb salvage procedure options was performed. The patient's parents were informed that data and images concerning the case would be submitted for publication, and they provided written informed consent.

## 2 CASE REPORT

**FIGURE 1:**
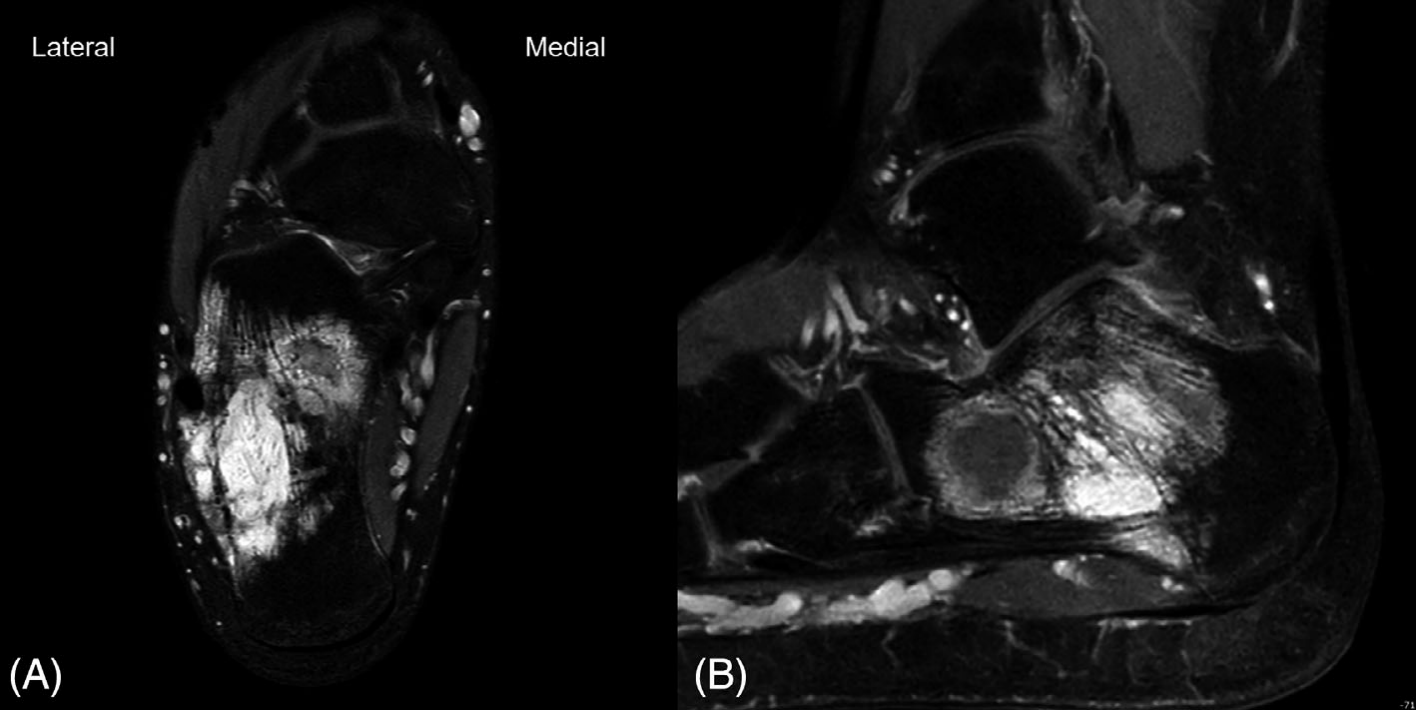
Magnetic resonance imaging with a calcaneus bone lesion with soft tissue component, very vascular, that breaks the bone cortex, with necrosis focus inside. (A) Coronal view. (B) Sagittal view

A 13‐year‐old male was admitted to our outpatient clinic with a 6‐month history of progressive right ankle swelling and weight bearing pain. Physical examination showed a swollen ankle with mild tenderness and slightly decreased range of motion. No fever or weight lost was found. Radiography and computed tomography (CT) revealed an expansive lytic lesion in the calcaneum with cortical break and no associated periosteal reaction. Magnetic resonance imaging (MRI) of the ankle showed a well‐defined bony lesion, isointense to T1‐weighted and heterogeneously hyperintense to T2‐weighted images, associated with a soft tissue mass. The tumor showed intense enhancement after contrast administration due to its high vascularity and also presented internal foci of necrosis (Figure 1A,B). CT and MRI did not show any involvement of the talus, navicular bone, distal tibia, or distal fibula. CT of the chest, Tc99 bone scan, and bone marrow examination excluded the presence of distant metastases.

Ultrasound‐guided biopsy was performed. Histologically biopsy showed a malignant tumor composed of uniform small round cells, diffusely distributed. The cells showed strong membranous immunohistochemical expression of CD99. Molecular study revealed a *EWSR1‐FLY1* fusion gene by RT‐PCR. A limb salvage surgery with a total calcanectomy and reconstruction with allograft calcaneus was planned since the calcaneus was almost completely involved, and there was no evidence of metastasis. The patient was admitted at the oncology ward and started on chemotherapy following the Practice Guidelines based on the Spanish sarcoma group 21 protocol (GEIS‐21).^19^ Surgery was performed after three cycles of induction chemotherapy with vincristine, doxorubicin, cardioxane, cyclophosphamide, ifosfamide, and etoposide. After recovering from surgery chemotherapy was restarted. Protocol regimen consisted of cycles 1, 2 and 4 with cyclophosphamide 4.2 g m^−2^, doxorubicin 75 mg m^−2^ and vincristine 2 mg m^−2^; and cycles 3 and 5 with ifosfamide 9 g m^−2^ and etoposide 500 mg m^−2^.

### 2.1 Surgical procedure

**FIGURE 2:**
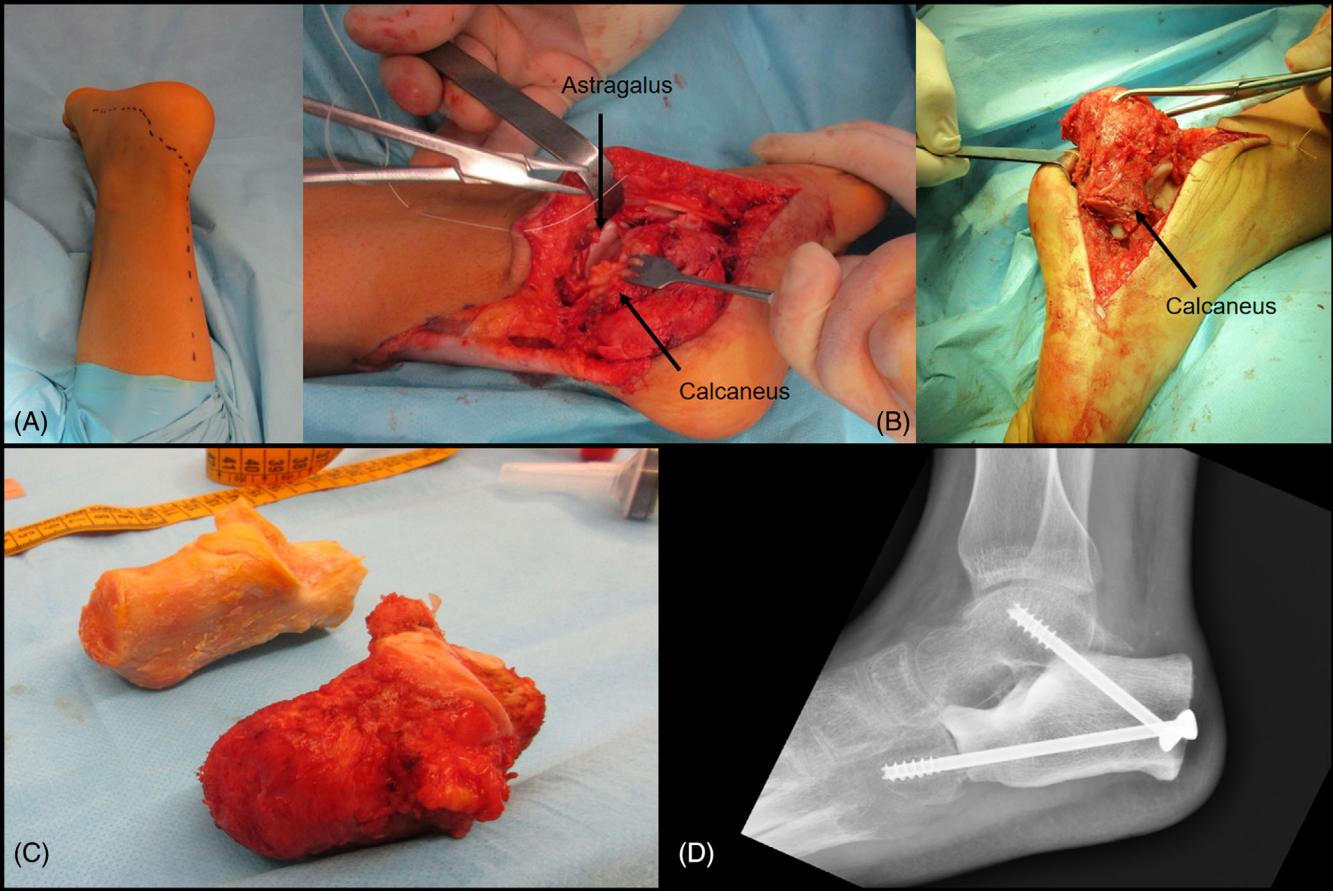
(A) Lateral calcaneus approach extended with a posterior ankle approach. (B) Dissection of the soft tissues. (C) Calcaneus resected in bloc. (D) Talocalcaneal and calcaneocuboid arthrodesis

The surgery was performed by specialist's pediatric oncologic orthopedic surgeons. Under spinal/epidural anesthesia, the patient was set in supine position with affected thigh elevated and pressurized by tourniquet. A lateral calcaneus approach extended with a posterior‐medial ankle approach was used to expose the subtalar joint and performed the total calcanectomy, avoiding the sural nerve and fibular tendons (Figure 2A). After the dissection of the soft tissues the calcaneus was bloc resected (Figure 2B,C). Then the subtalar cartilage of the astralagus and cuboid was removed to facilitate arthrodesis, and the calcaneus allograft was inserted and fitted to the calcaneus and cuboid. Under fluoroscopic control, the graft was fixed to the talus and the cuboid by two cannulated titanium screws, aiming to obtain a construct resembling a talocalcaneal and calcaneocuboid arthrodesis (Figure 2D). Afterward, Achilles tendon reintegration was performed by trans‐osseous points and a posterior suropedic cast with discrete equine was collocated. The surgery took approximately 120 minutes, and intraoperative blood loss was 50 ml.

The average size of the calcaneus was taken from the images obtained by foot MRI and CT scan. The calcaneal allograft was selected for being similar to the original in shape and size, being the best option available in the bone bank of the Barcelona Tissue Bank.

### 2.2 Histological findings and postoperative follow‐up

**FIGURE 3:**
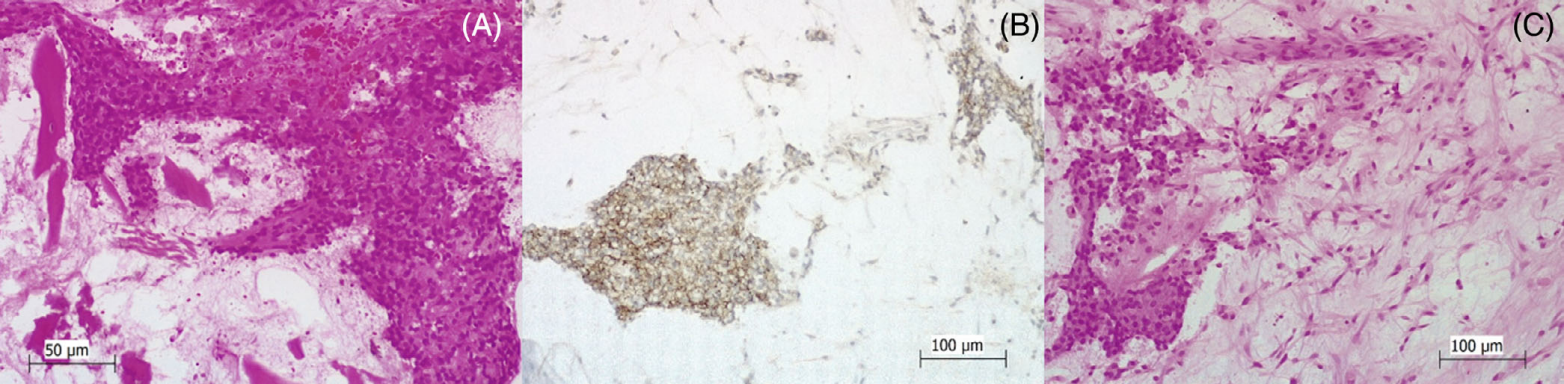
(A) Nests of tumor cells in a fibrous tissue background (*Hematoxylin and Eosin* [H & E], power of magnification 200×). (B) Tumoral cells of Ewing sarcoma showing membranous expression of CD99 (power of magnification 200×). Nest of tumoral small round cells (H & E; power of magnification 200×)

Pathological examination of the surgical specimen confirmed the calcaneus Ewing sarcoma with clear surgical margins, the closest ones were covered by periosteum, without infiltration, and soft tissue margins were of 5 mm (Figure 3A,B,C). There were no other perioperative complications. In the post‐operative period, a equinus cast was used for 4 weeks. After that, an orthosis with minimal equine was placed. Rehabilitation was started with progressive passive and active ankle movements without weight‐ bearing. Gradually, at 12 weeks after surgery, increased weight bearing was initiated and at 6 months, full weight‐bearing was reached. Our patient continued on chemotherapy according to protocol. Radiotherapy was not recommended.

**FIGURE 4:**
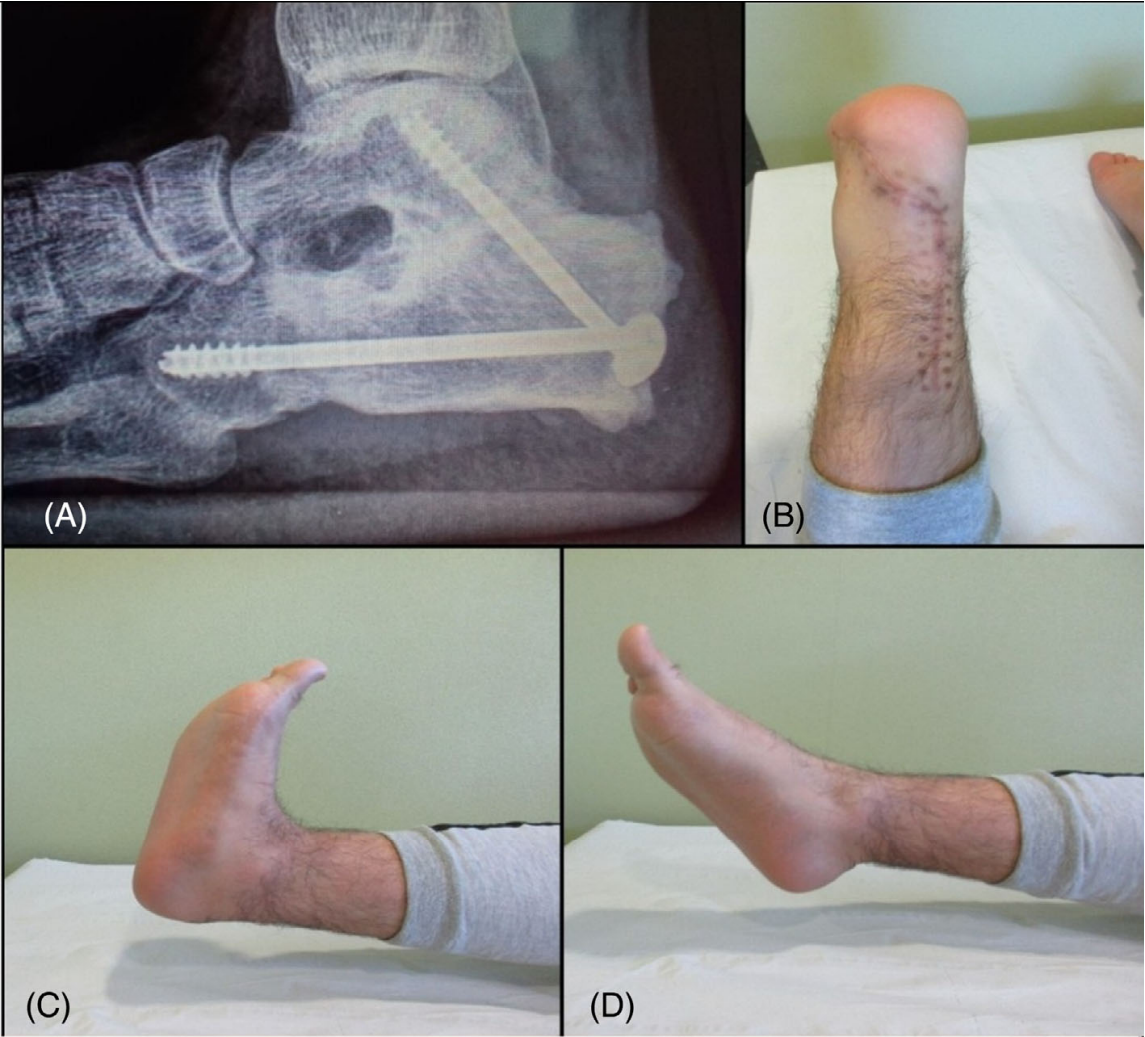
(A) X‐ray at 42 months of follow‐up showed an allograft calcaneus and screws in stable position, with arthrodesis healed successfully, especially at talocalcaneal joint. (B) Wound healing at final follow up. (C) Dorsiflexion of the ankle. (D) Plantar flexion of the ankle

Follow‐up radiography at 12 months showed normal position of the allograft and screws, with progressing healing of the arthrodeses (graft to talus and graft to cuboid) (Figure 4A). Follow‐up radiography at 42 months after surgery, the patient remained disease free, and he could weight bear and run almost normally without any associated symptoms. Dorsiflexion and plantar flexion were 15° and 30°, respectively (Figure 4B,C,D). Our patient recovered almost full range of motion and complete mobility. He was able to run and play soccer again. Musculoskeletal tumor society scoring system (MSTS) was 29 (pain = 5, function = 4, emotional = 5, support = 5, walking = 5, gait = 5).

## 3 DISCUSSION

With a conservative surgical management of a malignancy, it is difficult to achieve both adequate surgical margins and a functional reconstruction.^6^, ^9^, ^20^ Below‐knee amputation has been the standard treatment for malignant calcaneus tumor, being a total excision of the calcaneus a treatment of choice in aggressive benign tumors and in selected cases of malignant tumors.^6^ In calcanectomy without reconstruction, it has been reported that patients can only walk 2 to 3 kilometers with pain. Also, many of these patients have equinus contracture and clawing of their toes.^11^ In our case report both CT and MRI did not show any involvement of the astralagus, navicular, distal tibia, or distal fibula. Also, chest CT, Tc99 bone scan and bone marrow examination ruled out the presence of distant metastases. Despite Ewing Sarcoma being a malignant tumor, we considered performing a limb salvage surgery with reconstruction of the calcaneus. In our case the MRI revealed a well‐defined calcaneus bone lesion with soft tissue component that breaks the bone cortex. It has been studied that in these cases, although the cortex is involved, the anatomical characteristics of the calcaneus make limb salvage possible.^5^, ^20^ In the calcaneus only one aspect of the bone is close to the major neurovascular structure. Depending on the size and location of the extraosseous part, free surgical margins can be achieved by total calcanectomy along with the extraosseous part without sacrifice of the major neurovascular structure, which is important to maintain foot function.^5^, ^6^, ^20^

In our patient, a reconstruction with a frozen calcaneus after the total calcanectomy for the Ewing's sarcoma was performed. Reconstructive techniques for the hindfoot after total calcanectomy include osteocutaneous distally pedicled, free flap pedicled, prosthesis and allograft^11^, ^12^, ^18^ (Table 1). Pedicled osteocutaneous fibular flap has been successfully used for hindfoot reconstruction after traumatic total calcanectomy specially. Li et al. published their results using vascularized fibular flaps in combination with and without massive allografts.^15^, ^16^ Also, Innocenti et al. in 2019 published their results of heel reconstruction using an iliac crest free flap.^18^ Despite their good outcome reported, both techniques are microsurgical procedures that have technical difficulties and longer operation times. Moreover, both techniques have been used in total grown persons, more than 18 years old. A custom‐made prosthesis reconstruction for sarcomas of the calcaneus was first reported by Chou et al. in a 31 year old patient.^11^ In that report the prosthesis was fixed to the talus via a posterior approach including plantar skin incision. At the 12‐year follow‐up, the patient could not walk more than 8 to 10 blocks and had persistent pain in the plantar heel pad. Walking bare‐ foot was not possible, and the operated foot required a different shoe.^12^ Despite these options, in our case, we used a reconstruction with a frozen calcaneus because our patient was a child and we wanted to avoid a plantar skin incision.

**TABLE 1:** Review of the literature of different options of reconstruction after a calcanectomy for tumors

Study	Age	Histopathology	Reconstruction	Follow‐up	Complications
Li et al.^16^	43	Chondrosarcoma	Pedicled fibular flap and arthrodesis	6 years	No
	37	Chondrosarcoma	Pedicled fibular flap and arthrodesis	5 years	Hematoma. Require a hematoma evacuation
	19	Osteosarcoma	Pedicled fibular flap and arthrodesis	4 years	No
	16	Ewing sarcoma	Pedicled fibular flap and arthrodesis	3 years	No
	23	Ewing sarcoma	Pedicled fibular flap and arthrodesis	3 years	Skin margin necrosis. Skin graft
Li et al.^15^	27	Osteoblastoma	Allograft + pedicled fibular flap and arthrodesis	3 years	Wound infection. Require a debridement and drainage.
Kurvin et al.^14^	27	Osteosarcoma	Vascularized Iliac Bone Graft and arthrodesis	6 months	No mentioned
Scoccianti et al.^17^ Innocenti et al.^18^	18	Osteoblastoma	Vascularized Iliac Bone Graft and arthrodesis	19 years	Fracture of the Bone graft (At 6 months after surgery). No reoperation.
	29	Osteoblastoma	Vascularized Iliac Bone Graft and arthrodesis	17 years	Broke of one of the anterior screws (from cuboid to graft). No reoperation.
Innocenti et al.^18^	18	Ewing sarcoma	Vascularized Iliac Bone Graft and arthrodesis	9 years	Wound dehiscence (healed by negative pressure wound therapy). No reoperation.
	42	Osteosarcoma	Vascularized Iliac Bone Graft and arthrodesis	6 years	Wound dehiscence (healed by secondary intention). No reoperation.
Chou et al.^11^, ^12^	31	Osteosarcoma	Prosthesis and arthrodesis	12 years	Shoe limitation Moderate persistent pain Interfragmentary screws broke.
Imanishi et al.^9^	71	Chondrosarcoma	3D‐printed titanium calcaneal prosthesis	5 months	No mentioned
Ottlenghi et al.^7^ Muscolo et al.^8^	14	Chondrosarcoma	Total calcaneus allograft and arthrodesis	32 years	Grafted bone collapse. No reoperation
Muscolo et al.^8^	41	Giant cell tumor	Total calcaneus allograft and arthrodesis	9 years	Grafted bone collapse. No reoperation
Woźniak et al.^13^	15	Chondrosarcoma	Total calcaneus allograft and arthrodesis	6 months	No mentioned
	15	Synovial sarcoma	Total calcaneus allograft and arthrodesis	6 months	No mentioned
	15	Ewing sarcoma	Total calcaneus allograft and arthrodesis	6 months	No mentioned

Reconstruction with frozen calcaneus allograft after a total calcanectomy for the malignant bone tumor has been reported previously. Ottolenghi and Petracchi^7^ and Muscolo et al.^8^ were the first to study the possibility of this technique, describing two cases. The first case was a 14 years old boy with a calcaneus chondrosarcoma and the second case was a 41 years old man with a calcaneus giant‐cell tumor. In both reports, osteointegration was successful with satisfactory functional results. However, the authors reported secondary osteonecrosis of the allograft during the follow‐up. Even the osteonecrosis at final follow‐up at 9 and 32 years respectively, both patients were able to walk without support and had no pain. Our patient at last follow‐up, was able to run and play soccer. Based on the long‐term results previously reported, this procedure may be a durable reconstructive option after total calcanectomy. Another important fact to take in to account is the damage that radiotherapy can cause to the allograft. In our case radiotherapy was not indicated due to the good response to chemotherapy and complete resection with negative margins.

In our patient, the calcaneus allograft was fixed in place with compression screws after the joint surfaces were resect, as needed, to obtain a proper fit. Because the rare of the use of total calcaneus allograft, no biomechanical studies of the mode of fixation have been made. Based on the current literature we preferred a double arthrodesis with screws. Degeorge et al. made the calcaneocuboid arthrodesis with a Blount's staple, however, they reported that their patient developed a varus of the hindfoot.^10^ We prefer to use compression screws, which seem to us to be a more solid and stable synthesis. The patient did not develop limb length inequalities or malalignment of the foot.

Ewing sarcoma generally arises from diaphysis of long bones and axial skeleton; however, rarely, the small bones in the feet including calcaneus can be affected. We think that in skeletally immature children, reconstruction with calcaneus bone allograft is a good alternative among several options that are available such as custom‐made prosthesis, fibular or iliac crest flaps. We describe the use of this technique with a 42‐months follow‐up. At last follow‐up, our patient was disease free and had an excellent functional outcome. Further studies are needed to compare the available surgical options, including their functional outcome, complications and cost.

## 4 CONCLUSION

Total calcaneal allograft reconstruction of an Ewing's calcaneus sarcoma in a child is a good alternative limb salvage surgical treatment among several options that are available such as custom‐made prosthesis, fibular flaps, or iliac crest flaps.

## AUTHOR CONTRIBUTIONS

**Ferran Torner:** Conceptualization (lead); investigation (equal); methodology (equal); supervision (equal); validation (equal); writing – review and editing (equal). **Jorge H. Nuñez:** Conceptualization (equal); investigation (equal); methodology (equal); writing – original draft (equal). **Emilio José Inarejos Clemente:** Investigation (equal); supervision (equal); writing – review and editing (equal). **Moira Garraus:** Investigation (equal); methodology (equal); supervision (equal); validation (equal); writing – review and editing (equal). **Mariona Suñol:** Conceptualization (equal); investigation (equal); methodology (equal); supervision (equal); validation (equal); writing – review and editing (equal). **Aníbal D. Martínez:** Conceptualization (equal); investigation (equal); methodology (equal); resources (equal); supervision (equal); writing – review and editing (equal). **David Moreno:** Conceptualization (equal); investigation (equal); methodology (equal); supervision (equal); validation (equal); writing – review and editing (equal).

## CONFLICT OF INTEREST

The authors have stated explicitly that there are no conflicts of interest in connection with this article.

## ETHICS STATEMENT

This material is the authors' own original work, which has not been previously published elsewhere. The study was performed in accordance with the ethical standards aslaid down in the 1964 Declaration of Helsinki.

## Data Availability

Data sharing is not applicable to this article as no new data were created or analyzed in this study.
